# Factors influencing the trans-membrane transport of *n*-octadecane by *Pseudomonas* sp. DG17

**DOI:** 10.1080/13102818.2014.923601

**Published:** 2014-08-26

**Authors:** Fei Hua, Hong Qi Wang, Yi Cun Zhao

**Affiliations:** ^a^ Department of Water Ecology and Environment, College of Water Sciences, Beijing Normal University, Beijing, China

**Keywords:** environmental factors, *Pseudomonas*, octadecane, trans-membrane transport, biodegradation

## Abstract

In soil bioremediation techniques, the trans-membrane transport of hydrocarbons across the cell membrane is a new and complex point of understanding the process of hydrocarbons biodegradation. In this study, the effect of different environmental factors, including substrate concentration, bacterial inoculums, pH, salinity, substrate analogues and nutrients, on the transport of [^14^C]*n*-octadecane by *Pseudomonas* sp. DG17 was investigated. The results showed that cellular [^14^C]*n*-octadecane levels increased along with the increase in the substrate concentration. However, the trans-membrane transport of [^14^C]*n*-octadecane was a saturable process in the case of equal amounts of inoculum (biomass). The highest concentration of accumulated [^14^C]*n*-octadecane was 0.51 μmol mg^−1^ ± 0.028 μmol mg^−1^ after incubation for 20 min. Meanwhile, the cellular *n*-octadecane concentration decreased along with the biomass increase, and reached a stable level. Acidic/alkaline conditions, high salinity, and supplement of substrate analogues could inhibit the transport of [^14^C]*n*-octadecane by *Pseudomonas* sp. DG17, whereas nitrogen or phosphorus deficiency did not influence this transport. The results suggested that trans-membrane transport of octadecane depends on both the substrate concentration and the microorganism biomass, and extreme environmental conditions could influence the biodegradation ability of microorganisms through inhibiting the transport of extracellular octadecane.

## Introduction

Bioremediation is considered to be a most cost-effective technique to remove or reduce petroleum contaminants in the environment compared with physical or chemical remediation techniques.[1] Meanwhile, the intensity of petroleum biodegradation is influenced by several environmental factors, such as temperature, oxygen, pH value, salinity, nutrients, concentration of contaminant, aqueous solubility and the concentration of microorganisms in the affected site.[2–4] On the other hand, different microorganisms adapt to different optimal environmental factors. Some can grow well under saline, acidic or alkaline conditions, or in a cold environment, while others cannot survive in extreme environments. Another factor that may also inhibit the biodegradation ability of bacteria in the soil are the low concentrations of N and P available for bacterial growth or the imbalance in the C:N:P ratio.[5] Therefore, some environmental conditions should be adjusted, for example, by improving aeration and correcting the moisture level and pH, to stimulate bioremediation.[6]

Moreover, the biodegradation of hydrophobic compounds is a complicated multistage process. Two steps, uptake and transport across the cell membrane, occur preferentially before the biodegradation process. For hydrophobic compounds, the uptake process can be enhanced if they are available in a dissolved, solubilized, or emulsified state.[3,7,8] In recent years, studies on the second step of the process have presumed that these petroleum compounds may enter microorganism cells through passive diffusion, facilitated diffusion and/or active transport mechanisms.[9–12] The limitations connected with low aqueous solubility and inadequate transport are related to the molecular weight of the compound.[3] Depending on the arrangement of atoms, small-molecule compounds may easily traverse cellular membranes. Then, they are biodegraded by cellular oxygenases, converted into more easily biodegradable intermediate products or CO_2_.[13,14]

Many studies have reported how to isolate the optimal environment factors for petroleum biodegradation. However, the effect of environmental factors on the trans-membrane transport of hydrophobic alkanes, as an important stage of biodegradation, has been less studied. The aim of the present study was to show the effect of physical environmental factors on the trans-membrane transport of octadecane in *Pseudomonas* sp. DG17 isolated from soil polluted with crude oil. To that purpose, biodegradation experiments using non-labelled octadecane and trans-membrane transport experiments using ^14^C-labeled octadecane were conducted under aerobic conditions, varying the following parameters: pH, salinity, substrate concentration, biomass content, substrate analogue, nitrogen and phosphorus content.

## Materials and methods

### Culture and growth conditions

*Pseudomonas* sp. DG17 strain (China General Microbiological Culture Collection: CGMCC No. 5051; National Center for Biotechnology Information: NCBI accession No. JN 216879) used in this study was previously isolated from crude oil contaminated soils. According to Hua and Wang,[15] cells of DG17 were inoculated in mineral salt medium (MSM) sterilized by autoclaving at 121 °C for 20 min. MSM (pH 7.0) contained 0.4 g/L Na_2_HPO_4_, 0.15 g/L KH_2_PO_4_, 0.1 g/L NH_4_Cl, 0.05 g/L MgSO_4_·7H_2_O, 0.0015 g/L CaCl_2_, 0.1 g/L NaNO_3_, 1 mL trace elements solution (5 mg/L CuSO_4_·5H_2_O, 10 mg/L H_3_BO_3_, 10 mg/L MnSO_4_·5H_2_O, 70 mg/L ZnSO_4_). Cultures were maintained at 4 °C on crude oil solid medium and transferred monthly. Radiolabeled [^14^C]*n*-octadecane (ARC 1261, 99% pure, 55 mCi mmol^−1^) obtained from American Radiolabeled Chemicals, Inc. (St. Louis, Missouri, U.S.A.) was used as a sole carbon source. All glassware was treated with nitric acid to minimize the adsorption of octadecane.

### Concentrations of *n*-octadecane

Trans-membrane transport assay of *n*-octadecane by *Pseudomonas* sp. DG17 was performed by measuring the cellular [^14^C]*n*-octadecane in the presence of initial substrate concentrations ranging from 1.52 to 36.36 μmol L^−1^ (1.52, 4.54, 9.09, 18.18 and 36.36 μmol L^−1^). Methanol solution of [^14^C]*n*-octadecane (25 μCi mL^−1^) was added to 100 mL flasks containing 15 mL of cell suspension in MSM at room temperature. The initial cell biomass was 12 μg mL^−1^. Samples were collected at 1, 3, 5, 8, 11, 14, 17 and 20 min. At each time point, 1 mL aliquots were taken from the flask and applied to a Whatman GF/C glass fibre filter under vacuum. The cells in the filter were washed six times with 1 mL of phosphate buffer under vacuum before being transferred into 2 mL of scintillation fluid (nonylfenolethoxylate, 9016-45-9, PerkinElmer Waltham, Massachusetts, USA). The radioactivity was measured by PerkinElmer liquid scintillation counter (WallacOy 1450 Micro Beta). This fraction of [^14^C]*n*-octadecane was taken as cellular *n*-octadecane (μmol mg^−1^). Three samples were analysed at the same time for standard deviation analysis. During the incubation time, no significant loss of radioactivity due to volatilization of *n*-octadecane was observed.

### Inoculation amount of *Pseudomonas* sp. DG17

To analyse the transport of *n*-octadecane by *Pseudomonas* sp. DG17 under different cell densities, the following cell densities at 600 nm (A_600_) were applied: 0.1, 0.2, 0.5, 0.8, 1.0, 1.5 and 2.0. Accordingly, the biomass of DG17 cells was equivalent to 1.36, 4.24 13.11, 23.53, 30.88, 41.64 and 49.76 μg mL^−1^ protein, respectively. Methanol solution of [^14^C]*n*-octadecane (25 μCi·mL^-1^) was added to 100 mL flasks containing 15 mL of MSM. The final concentration of [^14^C]*n*-octadecane in the assay mixture was 1.52 μmol L^−1^. Assays were started when different amounts of cells were added in the medium. Samples were collected at 1 and 20 min. The concentration of cellular *n*-octadecane (μmol mg^−1^) was measured as described above.

### Substrate analogues of *n*-octadecane

To investigate the effect of substrate analogues on the trans-membrane transport of *n*-octadecane, 30 μL of methanol solution of [^14^C]*n*-octadecane (25 μCi mL^−1^) was supplemented into 100 mL flasks containing 15 mL of MSM and incubated at 30–35 °C for 20 min. The final concentration of [^14^C]*n*-octadecane in the assay mixture was 0.45 μmol L^−1^. The flasks were supplemented with 0.45 μmol L^−1^ of non-labelled *n*-octadecane, *n*-hexacosane, and *n*-triacontane before the addition of [^14^C]*n*-octadecane, respectively. Transport assay was initiated by the addition of DG17 cells. The initial cell density was 15 μg mL^−1^. Samples were collected at 1, 3, 5, 8, 11, 14, 17 and 20 min. The concentration of cellular *n*-octadecane was measured as described above.

Other hydrocarbons were also added as substrate analogues. The solvent for *n*-decane, *n*-dodecane, *n*-tetradecane, *n*-hexadecane, *n*-octadecane, *n*-tetracosane and *n*-hexacosane was hexane, the solvent for *n*-triacontane, *n*-dotriacontane and *n*-hexatriacontane was toluene, and the solvent for naphthalene and phenanthrene was acetone. In parallel, a *Pseudomonas* sp. DG17 culture was grown in medium that did not contain substrate analogues was taken as a control group.

Stock solutions of these substrate analogues were added in 50 mL tubes containing 4 mL of MSM, and the final substrate analogue concentration was 4.54 μmol L^−1^. After the solvent was volatilized, methanol solution of [^14^C]*n*-octadecane (25 μCi mL^-1^, 454.54 μmol L^−1^) was added to tubes with ^14^C concentration of 1.52 μmol L^−1^. Transport assay was started when DG17cells were added to the tubes. The initial cell density was 15 μg mL^−1^. Samples were collected at 1 and 20 min, respectively.

### Salinity and pH

The pH of MSM was adjusted to 5.42, 6.38, 7.43 or 9.72 with 2 mol L^−1^ of HCl or 2 mol L^−1^ of NaOH. NaCl (30%) stock solution was added to 100 mL flasks containing 60 mL of cell suspension in MSM at room temperature, and the final salinity was 0.5%, 1%, 3% and 5%. The initial cell density was 18 μg mL^−1^. Trans-membrane transport assay was started when methanol solution of [^14^C]*n*-octadecane (25 μCi mL^−1^) was added to the flasks, and the final [^14^C]*n*-octadecane concentration was 0.23 μmol L^−1^. Samples were collected at 1, 3, 5, 8, 11, 14, 17 and 20 min. The concentration of cellular *n*-octadecane (μmol mg^−1^) was measured as described above.

### Nutrient N and P

Three groups of assays were designed to investigate the influence of nutrient N and P on the transport process of *n*-octadecane by *Pseudomonas* sp. DG17. The content of mineral salt medium was different from that described above. In the first group, MSM contained Na_2_HPO_4_, KH_2_PO_4_ and NaNO_3_; in the second group, MSM did not contain nutrient nitrate NaNO_3_ and NH_4_Cl; in the third group, MSM did not contain nutrient Na_2_HPO_4_ and KH_2_PO_4_. Then, 12 μL of methanol solution of [^14^C]*n*-octadecane (25 μCi mL^−1^) was added to 100 mL of flasks containing 15 mL of MSM and incubated at 30–35 °C for 20 min. The final concentration of [^14^C]*n*-octadecane in the assay mixture was 0.36 μmol L^−1^.Trans-membrane transport was initiated by the addition of DG17 cells. The initial cell density was 15 μg mL^−1^. Samples were collected at 1, 3, 5, 8, 11, 14, 17 and 20 min, respectively. The concentration of cellular *n*-octadecane (μmol mg^−1^) was measured as described above.

## Results and discussion

Many factors can influence the bioremediation process and should be monitored. These factors include temperature, concentration of the pollutant, nutrient, oxygen availability and the biomass of microorganisms.[6] As one of the most important physical process before intracellular biodegradation starts, the transport of hydrocarbons across the microorganism cell membrane would also be influenced in the environment. Our experiments were designed to study the effect of several different factors on the uptake of [^14^C]*n*-octadecane by *Pseudomonas* sp. DG17. This strain was previously isolated from petroleum-contaminated soil and was shown to have good ability to pseudosolubilize C_12_ to C_28_ of *n*-alkanes during crude oil biodegradation process.[11]

### Effect of substrate concentrations on *[* ^14^C*]*n-octadecane transport

**Figure 1.**
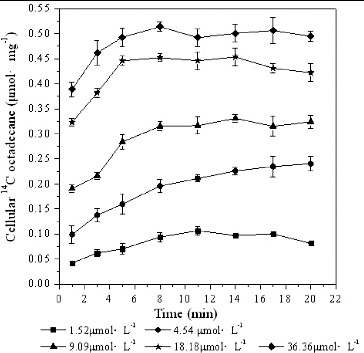
Cellular [^14^C]*n*-octadecane in *Pseudomonas* sp. DG17 in the presence of different substrate concentrations. The initial cell density was 12 μg mL^−1^. Standard deviations were less than 0.026 μmol mg^−1^.

Trans-membrane transport of *n*-octadecane by *Pseudomonas* sp. DG17 in the presence of different substrate concentrations is shown in Figure 1. The transport of extracellular [^14^C]*n*-octadecane proceeded at a high rate. For instance, the cellular ^14^C concentration in the samples taken at the first time point (1 min after initiation of the assay) was 0.042 ± 0.0029 μmol mg^−1^, 0.099 ± 0.018 μmol mg^−1^, 0.19 ± 0.0080 μmol mg^−1^, 0.32 ± 0.0081 μmol mg^−1^, 0.39 ± 0.014 μmol mg^−1^, respectively, at the addition of 1.52, 4.54, 9.09, 18.18 and 36.36 μmol L^−1^ of [^14^C]*n*-octadecane. Accordingly, the fractions of cellular ^14^C accounting for the total ^14^C were 33.13%, 26.17%, 25.17%, 21.32%, and 12.83%, respectively. Meanwhile, it was found that accumulated ^14^C increased the higher the initial substrate concentration was. For example, after incubation for 11 min, cellular [^14^C]*n*-octadecane reached 0.49 ± 0.016 μmol mg^−1^ at the addition of 36.36 μmol L^−1^ of [^14^C]*n*-octadecane. Accordingly, the fractions of cellular ^14^C accounting for total ^14^C were 84.38%, 55.68%, 41.76%, 29.44% and 16.24%, respectively. The cellular ^14^C concentrations increased to a steady level after incubation for 11 min. It could be inferred that an active transport mechanism existed in this process when the substrate concentration was lower than 9.09 μmol L^−1^. On the other hand, the intracellular concentration of *n*-octadecane was always lower than the extracellular one when the substrate concentration was above 9.09 μmol L^−1^. The highest amount of cellular [^14^C]*n*-octadecane was 0.51 ± 0.028 μmol mg^−1^ in the presence of 36.36 μmol L^−1^ extracellular substrate. Although the initial transport of *n*-octadecane was quick (within 1 min) and could be accelerated under higher substrate concentrations, an important observation from our study is that the accumulation of *n*-octadecane was a saturable process. The cellular substrate increased to a stable level in the case of equal amounts of initial inoculum and would not increase further even under excessive substrate concentration.

In the report of Ferreira et al., [2] for example, the initial cell concentration had a positive effect, whereas the initial petroleum concentration had a negative effect on the crude oil removal. Similarly, an excessively high hydrocarbon concentration could decrease the degradation ability of the bacterium *Bacillus fusiformis* sp. KL2-13 even when the other cultivation conditions are set at optimum values. The hydrocarbon degradation rate reached 59.07% ± 0.37% under the optimum culture conditions: pH range of 6–8, inoculum quantity of 3% and substrate dosage of 2%. [16] According to Towell et al., [17] cable oil concentration had a significant effect upon oil biodegradation. Microbial respiratory activity increased with increasing cable oil concentration, whereas [^14^C]phenydodecane mineralization decreased. Cable oil biodegradation was a function of cable oil concentration and catabolic ability of microbial populations.

It is known that contaminant concentrations can directly influence microbial activity. On the one hand, when they are too high, the contaminants may have toxic effects on the present bacteria. On the other hand, low contaminant concentrations may prevent the induction of bacterial degradation enzymes. In our study, during the *n*-octadecane transport process, cellular ^14^C levels increased with time and reached a stable level. Meanwhile, it was found that high concentration of substrate could encourage the transport of extracellular *n*-octadecane. However, in the case of the same amount of biomass inoculum, the trans-membrane transport of the substrate was also limited, for no more cells could still accumulate extracellular octadecane. Then, in the case of low substrate concentration, the trans-membrane transport of [^14^C]*n*-octadecane was also limited even though the cellular substrate might have not been saturated. Thus, it was demonstrated that the effect of substrate concentration on the transport of extracellular octadecane was complex. These results are in agreement with some biodegradation effects observed in other reports. For example, Goldstein et al.[18] suggested low concentrations as one likely reason for failure of inoculation with degrading microorganisms to enhance mineralization of phenolic compounds in soil and water samples. Wiggins and Alexander [19] also indicated that low contaminant concentration was one factor that lengthened the acclimation period before mineralization of different xenobiotic compounds by indigenous microbial communities.

### Effect of inoculum on *[* ^14^C*]* *n*-octadecane transport

**Figure 2.**
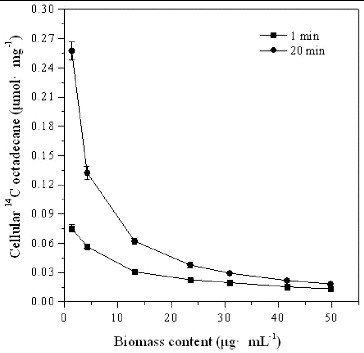
Cellular [^14^C]*n*-octadecane in *Pseudomonas* sp. DG17 in the presence of different biomass content. Standard deviations were less than 0.0094 μmol mg^−1^.

The effect of *Pseudomonas* sp. DG17 cell amount on the transport of [^14^C]*n*-octadecane was also analysed. As shown in Figure 2, the initial cellular [^14^C]*n*-octadecane was 0.076 μmol mg^−1^ ± 0.0034 μmol mg^−1^, 0.057 μmol mg^−1^ ± 0.0017 μmol mg^−1^, 0.031 μmol mg^−1^ ± 0.0015 μmol mg^−1^, 0.019 μmol mg^−1^ ± 0.0012 μmol mg^−1^, and 0.013 μmol mg^−1^ ± 0.00067 μmol mg^−1^, respectively, in the addition of cells equivalent to 1.36, 4.24, 13.11, 30.88 and 49.77 mg L^−1^ protein. With time, the cellular [^14^C]*n*-octadecane concentration increased along with the increase of cell biomass, and reached a stable level of 0.018 μmol mg^−1^ ± 0.0011 μmol mg^−1^ after incubation for 20 min. This result indicated that the ability of *Pseudomonas* sp. DG17 to accumulate the substrate was limited by the extracellular substrate concentration. This suggested that cellular [^14^C]*n*-octadecane was not increased along with the increase of cell amount. The biodegradability of the substrate was limited, although the biomass of *Pseudomonas* sp. DG17 in the environment was sufficient.

On the other hand, it was found that cellular [^14^C]*n*-octadecane declined along with the increase of DG17 biomass in the case of a fixed substrate concentration. In the condition of excessive biomass, transport of extracellular octadecane was also influenced, which means that some cells in the environment would not accumulate extracellular substrate. In our study, it was inferred that biomass did not have a negative effect on the transport of octadecane. However, the density of degrading microorganisms can play a role in the rate and extent of biodegradation. In this case, the inoculum size should also be considered as a factor that influences the effect of bioaugmentation of soil contaminated with crude oil.[20] Inoculation with an active population of degrading strains and making it work cooperatively with indigenous microorganisms is an option for enhancing the biodegradation rate of petroleum compounds.[21] For example, increasing the inoculum size of pentachlorophenol (PCP)-degrading strains *Arthrobacter* sp. [22] or *Flavobacterium* [23] obviously decreased the time necessary for 90% of the PCP to be degraded. PCP degradation rate was directly correlated with the inoculum size.

### Effect of substrate analogues on *[* ^14^C*]* *n*-octadecane transport

**Figure 3.**
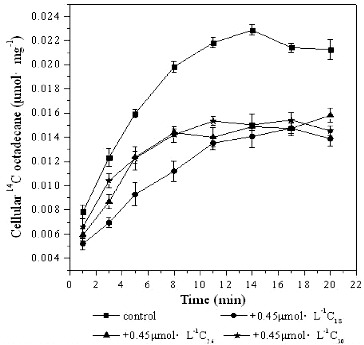
Cellular [^14^C]*n*-octadecane in *Pseudomonas* sp. DG17 in the presence of different non-labelled alkanes. The initial cell density was 15 μg mL^−1^. Standard deviations were less than 0.00097 μmol mg^−1^.

Petroleum contains different components, and microorganisms can uptake and utilize some kinds of these hydrophobic compounds simultaneously. To verify whether [^14^C]*n*-octadecane transport in DG17 cells is influenced by other analogues, excess of non-labelled *n*-octadecane, *n*-hexacosane, and *n*-triacontane were supplemented in the culture medium with labelled *n*-octadecane. As shown in Figure 3, non-labelled alkanes inhibited the transport of cell-associated [^14^C]*n*-octadecane. For cells treated with labelled [^14^C]*n*-octadecane only, cellular [^14^C]*n*-octadecane reached 0.021 μmol mg^−1^ ± 0.00085 μmol mg^−1^. Meanwhile, cellular ^14^C levels in cells incubated with 0.45 μmol L^−1^ of [^14^C]*n*-octadecane plus 0.45 μmol L^−1^ of non-labelled *n*-octadecane, *n*-hexacosane or *n*-triacontane were lower: 0.014 μmol mg^−1^ ± 0.00062 μmol mg^−1^, 0.016 μmol mg^−1^ ± 0.00057 μmol mg^−1^, 0.014 μmol mg^−1^ ± 0.00039 μmol mg^−1^, respectively. This indicated that the transport of [^14^C]*n*-octadecane was immediately inhibited when non-labelled alkanes were added simultaneously. Labelled [^14^C]*n*-octadecane and non-labelled alkanes might be transported across the cell membrane or intercalated in the membrane at the same time.

The other petroleum hydrocarbons tested, including decane, triacontane, naphthalene, phenanthrene, etc., also showed an inhibiting effect on [^14^C]*n*-octadecane transport. As shown in Table 1 cellular [^14^C]*n*-octadecane concentration increased to 0.061 μmol mg^−1^ ± 0.0084 μmol mg^−1^ when only octadecane was added in the medium after incubation for 20 min. However, this value decreased from 0.0247 to 0.0387 μmol mg^−1^ when non-labelled substrate analogues were added in the medium. This result suggested that *Pseudomonas* sp. DG17 could transport different hydrocarbons into the membrane simultaneously, possibly in a competitive manner. It could be inferred that *Pseudomonas* sp. DG17 uses these different substrate analogues as carbon sources in the environment.

**Table 1.** Effect of non-labelled hydrocarbons on the trans-membrane transport of [^14^C]*n*-octadecane by *Pseudomonas* sp. DG17.

Hydrocarbons	1 min (μmol mg^−1^)	20 min (μmol mg^−1^)
Control	0.0433 ± 0.0023	0.0607 ± 0.0084
*n*-decane	0.0193 ± 0.0014	0.0220 ± 0.0006
*n*-dodecane	0.0200 ± 0.0011	0.0233 ± 0.0014
*n*-tetradecane	0.0193 ± 0.0005	0.0260 ± 0.0076
*n*-hexadecane	0.0193 ± 0.0013	0.0233 ± 0.0039
*n*-octadecane	0.0207 ± 0.0021	0.0240 ± 0.00085
naphthalene	0.0213 ± 0.0008	0.0360 ± 0.0014
phenanthrene	0.0273 ± 0.0015	0.0287 ± 0.0009
*n*-tetracosane	0.0253 ± 0.0016	0.0287 ± 0.0017
*n*-hexacosane	0.0273 ± 0.0020	0.0300 ± 0.0012
*n*-triacontane	0.0293 ± 0.0007	0.0320 ± 0.0028
*n*-dotriacontane	0.0293 ± 0.0008	0.0333 ± 0.0025
*n*-hexatriacontane	0.0327 ± 0.0015	0.0340 ± 0.0019

Note: Concentration of [^14^C]*n*-octadecane was 1.52 μmol L^−1^. Non-labelled alkane was 4.54 μmol L^−1^. The initial cell content was 15 μg mL^−1^. Standard deviations were less than 0.0084 μmol mg^−1^.

Similarly, for *Mycobacterium* sp. strain RJGII-135, addition of non-labelled phenanthrene at 0.15, 0.5 and 5.5 μmol L^−1^ reduced the cellular [^14^C]phenanthrene by 65%, 86% and 96%, respectively.[12] Meanwhile, anthracene, fluoranthene and pyrene reduced the ^14^C level to 48%, 46% and 43% of the control, respectively.[12] For *Arthrobacter* sp. strain Sphe3, when cells were incubated with 1.2 μmol L^−1^ of [^14^C]phenanthrene plus 1.2, 2.4 or 4.2 μmol L^−1^ of non-labelled phenanthrene, the cellular ^14^C level was reduced by 45%, 65% or 84%, respectively.[9] In this case, the substrate analogues do not have an inhibition effect on the transport function of microorganisms, but do block the transport of octadecane across the cell membrane.

### Effect of pH on *[* ^14^C*]* *n*-octadecane transport

**Figure 4.**
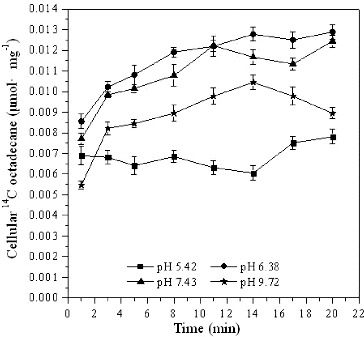
Cellular [^14^C]*n*-octadecane in *Pseudomonas* sp. DG17 at different pH. The initial cell density was 18 μg mL^−1^. Standard deviations were less than 0.00081 μmol mg^−1^.

The pH value affects the solubility and biological availability of nutrients, metals, and other constituents; for optimal bacterial growth, pH should remain within the tolerance range for the target microorganisms. Bioremediation processes preferentially proceed at a pH range of 6–9.[2] Severe changes of pH, such as strong acidic or alkaline conditions, damage the normal functions of membrane channel proteins, transporters and signalling pathway proteins, causing cells to lose the function of permselectivity. For the trans-membrane transport of *n*-octadecane by *Pseudomonas* sp. DG17, the optimum pH was around 7 (Figure 4). For cells grown at pH at 5.42 and 9.72, the cellular ^14^C levels were lower than those in cells grown at pH at 6.38 or 7.43. What is more, at pH 5.42 cellular [^14^C]*n*-octadecane levels did not increase considerably, which indicated that the transport of [^14^C]*n*-octadecane by *Pseudomonas* sp. DG17 was limited at this pH value. After incubation for 20 min, accumulated ^14^C increased to 0.0078 μmol mg^−1^ ± 0.00035 μmol mg^−1^, 0.013 μmol mg^−1^ ± 0.00034 μmol mg^−1^, 0.012 μmol mg^−1^ ± 0.00029 μmol mg^−1^, 0.0089 μmol mg^−1^ ± 0.00027 μmol mg^−1^ at pH values of 5.42, 6.38, 7.43 and 9.72, respectively. It was suggested that trans-membrane transport of octadecane by *Pseudomonas* sp. DG17 can be partly inhibited in acidic or alkaline environment. On the other hand, different microorganisms have been reported to be capable of biodegradation at extreme pH values. For example, Stapleton et al. [28] found that when the pH value of soil used as long-term storage piles was as low as 2, the indigenous microbiota in the soil could still oxidize toluene and naphthalene in the soil to CO_2_. Kanekar et al. [29] found that in Lonarlake, India, under the condition of pH up to 10, four kinds of indigenous microorganisms could still biodegrade pollutants such as methyl violet in wastewater discharged from a printing and dyeing mill.

### Effect of salinity on *[* ^14^C*]* *n*-octadecane transport

**Figure 5.**
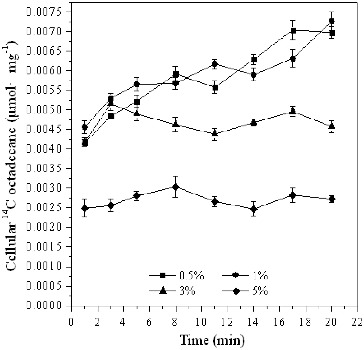
Cellular [^14^C]*n*-octadecane in *Pseudomonas* sp. DG17 at different salinity. The initial cell density was 28 μg mL^−1^. Standard deviations were less than 0.00027 μmol mg^−1^.

The results from our experiments on the transport of [^14^C]*n*-octadecane by *Pseudomonas* sp. DG17 under different salinity showed that high salinity could inhibit the initial trans-membrane transport of *n*-octadecane (Figure 5). For example, the cellular ^14^C concentration was only 0.0025 μmol mg^−1^ ± 0.00023 μmol mg^−1^ when the salinity of the medium was 5%, whereas this value was 0.0042 μmol mg^−1^ ± 0.00011 μmol mg^−1^ and 0.0046 μmol mg^−1^ ± 0.00016 μmol mg^−1^, respectively, when the salinity was 0.5% and 1%. The cellular ^14^C concentration did not increase at 3% and 5% salinity. After incubation for 20 min, this value increased to 0.0070 μmol mg^−1^ ± 0.00015 μmol mg^−1^, 0.0073 μmol mg^−1^ ± 0.00023 μmol mg^−1^, 0.0046 μmol mg^−1^ ± 0.00015 μmol mg^−1^, 0.0027 μmol mg^−1^ ± 0.000093 μmol mg^−1^, respectively, at 0.5%, 1%, 3% and 5% salt concentration. This indicated that the trans-membrane transport of octadecane was prevented in environments with high salinity.

High salinity is known to cause dehydration, which leads to severe changes of the internal environment and eventually to interruption of the biochemical reaction processes and cell death. In some cases, salt can react with the cell membrane and cause the membrane properties to change considerably. For the trans-membrane transport of *n*-octadecane by *Pseudomonas* sp. DG17, the optimum salinity range was between 0.5% and 1% (Figure 5). For example, the cellular ^14^C concentration was only 0.0025 μmol mg^−1^ ± 0.00023 μmol mg^−1^ when the salinity of the medium was 5%, whereas this value was 0.0042 μmol mg^−1^ ± 0.00011 μmol mg^−1^ and 0.0046 μmol mg^−1^ ± 0.00016 μmol mg^−1^, respectively, when the salinity was 0.5% and 1%. The cellular ^14^C concentration did not increase at 3% and 5% salinity. After incubation for 20 min, this value increased to 0.0070 μmol mg^−1^ ± 0.00015 μmol mg^−1^, 0.0073 μmol mg^−1^ ± 0.00023 μmol mg^−1^, 0.0046 μmol mg^−1^ ± 0.00015 μmol mg^−1^, 0.0027 μmol mg^−1^ ± 0.000093 μmol mg^−1^, respectively, at 0.5%, 1%, 3% and 5% salt concentration. This indicated that the trans-membrane transport of octadecane was inhibited in environments with high salinity. Similarly, Rhykerd et al. [26] found that the biodegradability of petroleum by indigenous microorganisms in the addition of 2% NaCl was lower compared with that in the addition of 0.4% or 1.2% (w/w) NaCl. However, in extreme conditions such as acidic/alkaline environments, or high-salinity environments, it was found that many kinds of microorganisms still have the potential to biodegrade petroleum. These microorganisms have high tolerance to extreme environments, and the transport of hydrocarbons is not influenced or inhibited. For example, acidophilic microorganisms can be used for bioremediation of soil polluted with heavy metals and organic compounds.[27] Halophilic microorganisms have been shown to be able to biodegrade and transform organic pollutants in environments where salinity is high.[30] Microbes need salt to maintain the balance of osmotic pressure, and the enzymes inside the cell membrane have to be able to adapt to the high-salinity environment to survive. For a NaCl-tolerant mutant of *Enterobacter cloacae*, rapid accumulation of K^+^ inside the cell and of extracellular polymeric substance outside the cell was observed when the mutant was cultivated in Luria–Bertani medium with 9.0% of NaCl. These factors contribute to maintenance of the membrane permeability and osmotic balance.[31]

### Effect of nutrients on *[* ^14^C*]* *n*-octadecane transport

**Figure 6.**
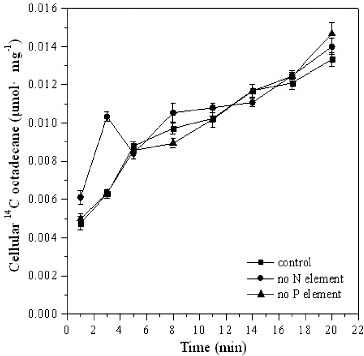
Cellular [^14^C]*n*-octadecane in *Pseudomonas* sp. DG17 in the lack of nutrients. The initial cell density was 15 μg mL^−1^. Standard deviations were less than 0.00057 μmol mg^−1^.

Nutrients, especially nitrogen, phosphorus, and in some cases iron, are very important ingredients for the successful biodegradation of hydrocarbon pollutants.[24] In most cases, supplement of nutrient such as N and P was helpful to reduce contaminants which could meet the biomass increase of biodegradable microorganisms.[5,25] In this study, however, deficiency of nutrient N or P did not seem to have an inhibiting effect on the transport of extracellular [^14^C]*n*-octadecane (Figure 6). The initial cellular ^14^C concentrations were 0.0061 μmol mg^−1^ ± 0.00035 μmol mg^−1^, 0.0050 μmol mg^−1^ ± 0.00025 μmol mg^−1^, 0.0048 μmol mg^−1^ ± 0.00036 μmol mg^−1^, respectively, in the absence of N, P, and in the presence of both N and P. The cellular ^14^C concentrations increased with time in all the variants and after incubation for 20 min, reached 0.014 μmol mg^−1^ ± 0.00042 μmol mg^−1^, 0.015 μmol mg^−1^ ± 0.00057 μmol mg^−1^ and 0.012 μmol mg^−1^ ± 0.00035 μmol mg^−1^, respectively.

It is well known that the accumulation of microorganism biomass is influenced in the absence of a nutrient. However, our experiments showed that the trans-membrane transport of octadecane was not blocked in the absence of N or P, indicating that N- or P-containing nutrients were not a factor that influences the trans-membrane transport of hydrocarbons in *Pseudomonas* sp. DG17. Taken together, all of these results indicated that the trans-membrane transport of octadecane was related to the biomass of *Pseudomonas* sp. DG17.

## Conclusions

In this study, laboratory studies were conducted to determine the effects of environmental factors on the trans-membrane transport of ^14^C-labeled *n*-octadecane. The results revealed that the ability of DG17 cells to accumulate octadecane was limited; and increasing the biomass or substrate under feasible conditions beyond the level of saturation did not lead to an increase in the cellular octadecane content. The highest amount of cellular [^14^C]*n*-octadecane was 0.51 μmol mg^−1^ ± 0.028 μmol mg^−1^ in the presence of 36.36 μmol L^−1^ of [^14^C]*n*-octadecane. *Pseudomonas* sp. DG17 could also simultaneously transport different hydrocarbon substrate analogues across the membrane, possibly in a competitive manner. Moreover, strong acidic, alkaline or high-salinity conditions could block the trans-membrane transport of the extracellular substrate. The optimal pH and salinity conditions for octadecane trans-membrane transport in *Pseudomonas* sp. DG17 were 6–8 and 0.5%–1%, respectively. Deficiency of nutrient N or P did not seem to have an inhibiting effect on the transport of extracellular [^14^C]*n*-octadecane, which suggested that nutrient deficiency could inhibit the biomass increase, and in turn, decrease the total substrate accumulated in the cells of *Pseudomonas* sp. DG17.
